# MiRNAs as Promising Translational Strategies for Neuronal Repair and Regeneration in Spinal Cord Injury

**DOI:** 10.3390/cells11142177

**Published:** 2022-07-12

**Authors:** Serena Silvestro, Emanuela Mazzon

**Affiliations:** IRCCS Centro Neurolesi “Bonino-Pulejo”, Via Provinciale Palermo, Contrada Casazza, 98124 Messina, Italy; serena.silvestro@irccsme.it

**Keywords:** spinal cord injury, microRNAs, neuronal repair, axonal regeneration

## Abstract

Spinal cord injury (SCI) represents a devastating injury to the central nervous system (CNS) that is responsible for impaired mobility and sensory function in SCI patients. The hallmarks of SCI include neuroinflammation, axonal degeneration, neuronal loss, and reactive gliosis. Current strategies, including stem cell transplantation, have not led to successful clinical therapy. MiRNAs are crucial for the differentiation of neural cell types during CNS development, as well as for pathological processes after neural injury including SCI. This makes them ideal candidates for therapy in this condition. Indeed, several studies have demonstrated the involvement of miRNAs that are expressed differently in CNS injury. In this context, the purpose of the review is to provide an overview of the pre-clinical evidence evaluating the use of miRNA therapy in SCI. Specifically, we have focused our attention on miRNAs that are widely associated with neuronal and axon regeneration. “MiRNA replacement therapy” aims to transfer miRNAs to diseased cells and improve targeting efficacy in the cells, and this new therapeutic tool could provide a promising technique to promote SCI repair and reduce functional deficits.

## 1. Introduction

Spinal cord injury (SCI) is a permanent injury that is caused by direct trauma to the tissue and is associated with the loss of motor, sensory, and autonomic functions [1]. According to the World Health Organization (WHO), about 250,000–500,000 people worldwide are affected by SCI [2]. The annual incidence is variable, for example in Denmark there are 9.2 cases per million, while in the United States it is 40.1 per million [3], with a worldwide incidence ranging from 3.6 to 195.4 per million [4]. SCI, in addition to affecting the physical and psychological health of the patient, also affects family members and is a burden in general for the community and the economy. SCI is caused by road accidents, violence, falls, and other common traumatic events [5]. When a continuous or instantaneous force is exerted, it induces the fracture or displacement of the spine with consequent compression or fracture of the spinal cord. These external forces that are responsible for physical tissue damage induce the primary injury. The primary lesion is followed by a series of physiological and biochemical reactions that lead to the secondary lesion. In this phase, several events such as edema, hematoma, and neuroinflammation occur at the site of the lesion [6]. Furthermore, the lesion leads to diffuse apoptosis in neurons, microglia, oligodendrocytes, and astrocytes [7]. Subsequently, the degree of injury extends causing neurological and locomotor dysfunction [8].

The complex cellular and molecular changes that arise after the injury are still poorly understood, making it difficult to develop resolutive treatments. Indeed, due to the limited regenerative capacity of the central nervous system (CNS), neuronal recovery after SCI is rare [9]. Primarily, treatment after SCI involves stabilization of the patient, prevention of complications, and physical rehabilitation. To date, the pharmacological treatment in use involves the administration of high-dose corticosteroids within the first 8 h after the injury and then continued therapy for 1–2 days [10]. Research over the past two decades has made numerous efforts to find regenerative strategies. Furthermore, a better understanding of the SCI pathophysiology and the number of advances that have been made in therapies involving the use of cells, biomaterials, and biomolecules has allowed the development of new therapeutic tools that are aimed at promoting neuroregeneration [11,12,13].

MicroRNAs (miRNAs) are single-stranded non-coding RNA molecules that bind target messenger RNA (mRNA) molecules interfering with translation mechanisms [14,15]. Several evidences have demonstrated the presence of abnormally expressed miRNAs in the plasma of SCI patients, thus suggesting the role of miRNAs as potential biomarkers in the diagnosis of SCI [16,17]. In addition to the role of miRNAs as biomarkers, numerous preclinical evidences have illustrated the role of miRNAs in SCI [18,19]. In an animal model of SCI, it was shown that miR-940 was decreased after injury, while miR-940 overexpression by inhibiting inflammation promoted functional recovery [20]. In another preclinical study, miRNA-124 was shown to suppress apoptosis of neuronal cells inducing functional recovery in rats with SCI [21]. Understanding the role of miRNAs in the pathophysiology of SCI appears of great scientific relevance and interest.

In this review, the pathophysiology of SCI will be illustrated detailing the potential role of miRNAs in the pathological process of this condition. Furthermore, the biogenesis and structure of miRNAs will be mentioned. We will provide an overview of miRNAs that are employed as potential therapeutic interventions after SCI, focusing our attention on miRNAs that are widely associated with neuronal and axonal regeneration.

## 2. Methodology

**Figure 1:**
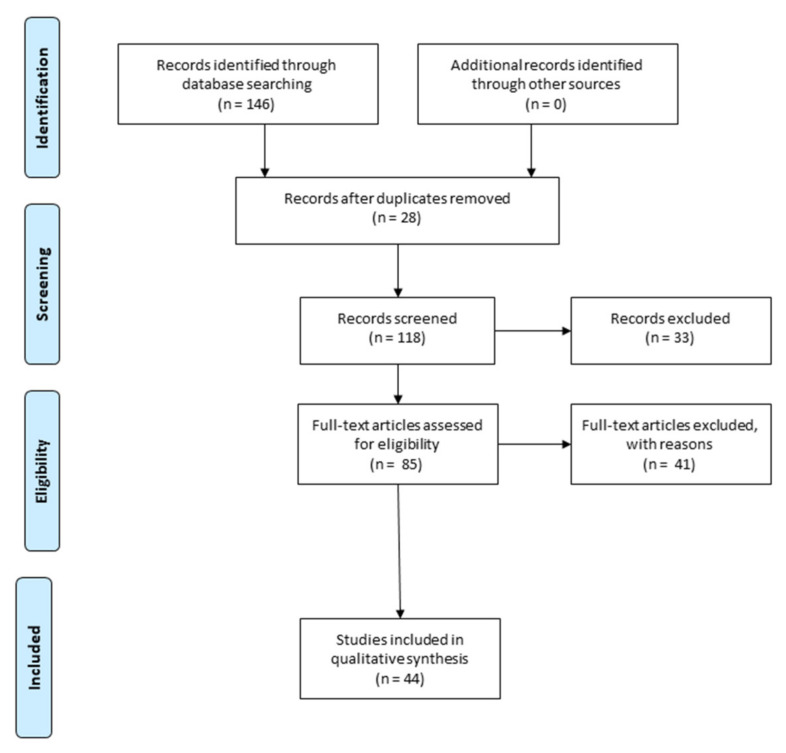
Prisma flow diagram describes the methodology that was employed to select the preclinical studies that were used for the writing of the review. Duplicate articles were excluded from the total of the studies that were recorded. Instead, we selected the articles that evaluate the microRNAs (miRNAs) that are widely related to neuronal and axon regeneration, important to promote spinal cord injury (SCI) repair, and reduce functional deficits (The PRISMA Statement is published in [22]).

In this review, the selected publications range from 2017 to 2022. In order to write the paragraphs “5. MiRNAs expression in SCI” and “6. MiRNAs for neuronal regeneration in SCI”, the bibliography research in PubMed was performed using the following keywords “spinal cord injury”, “microRNA biomarkers”, “microRNA”, “axonal regeneration”, and “neuronal regeneration”. We specifically selected the evidence that evaluated miRNAs that are widely associated with neuronal and axon regeneration, in order to promote SCI repair and reduce functional deficits, as illustrated in the Prisma flow diagram (Figure 1).

## 3. Pathophysiology of SCI

SCI is a complex medical and life condition. Historically, this disability was related to high mortality rates. SCI is associated with significant reductions in life expectancy that are based on injury severity and age at the time of the insult [23]. The available treatments are limited and generally provide only relief for these patients [24]. However, thanks to improvements in medicine, today SCI is no longer seen as the end of useful life but rather as a personal and social challenge that can be overcome.

In order to understand the pathophysiology of SCI, it is important to know the anatomy of the spinal cord. The spinal cord is located inside the vertebral column and extends from the brain to the vertebral level (L1-L2), ending in the medullary cone. The spinal cord itself has segmented neurological layers that correspond to the nerve roots exiting the spine in correspondence with the spaces between the vertebrae. There are 31 pairs of nerve roots: 8 cervical, 12 thoracics, 5 lumbar, 5 sacral, and 1 coccygeal [25].

SCI can be caused by traumatic or non-traumatic events. Traumatic SCI can be induced by multiple events such as falls, road accidents, injuries at work, and in sports, or acts of violence [26]. Non-traumatic SCI, instead, usually results from other pathologies such as infectious diseases, tumors, musculoskeletal diseases, and congenital problems such as spine bifida [27].

The symptoms of SCI depend on the extent of the injury and can cause sensory or motor loss of the lower limbs, trunk, and upper limbs, and the loss of autonomous (involuntary) body regulations. This can cause impaired breathing, alteration of heart rate, blood pressure, and temperature control, sexual dysfunction, and bowel and bladder loss of control [28,29].

The impairment degree is related to the level of the injury; in general, the higher the injury, the greater the disability degree. The cervical segments injury usually induce sensory and motor loss (paralysis) in the arms, body, and legs, known as tetraplegia [30]. A C4 or higher lesion directly interferes with autonomic control of breathing. An injury to the thoracic segments usually causes paraplegia, which is sensory and/or motor loss in the trunk and legs [31], while lumbar injury usually causes sensory and motor loss in the lower abdomen and legs [32].

According to the International Standards for Neurological Classification of SCI and the American Spinal Injury Association (ASIA), SCI can be classified as “complete” or “incomplete”. In the “complete” lesion, neurological assessments show no spared motor or sensory function below the lesion level. Conversely, in the “incomplete” lesion some motor and sensory functions below the lesion level are spared [33].

**Figure 2:**
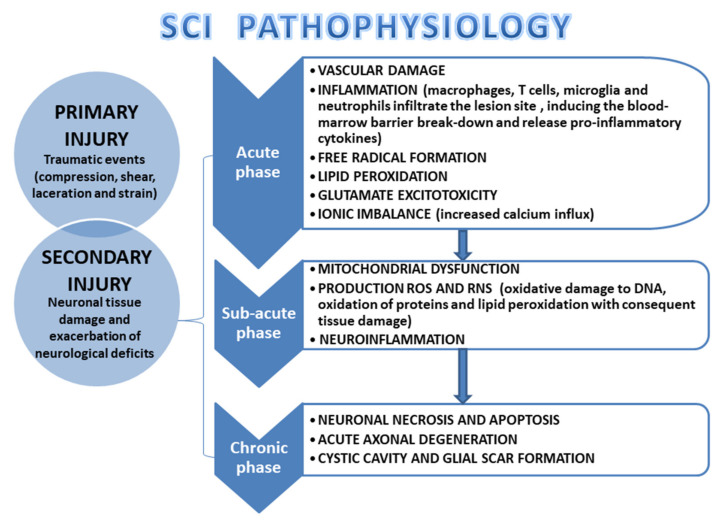
Schematic representation of SCI pathophysiology. Pathophysiology, clinical signs, and SCI phases.

Traumatic events are due to physical forces such as compression, shear, laceration, and strain can also cause SCI [34]. The extent of damage and the spinal cord compression duration are the most important determinants of the injury severity. This initial phase immediately following the injury is known as a “primary injury” which can be characterized by bone fragmentation and tearing of the spinal ligament [35]. This phase subsequently promotes a cascade of events that are involved in the progressive damage of the neuronal tissue and the exacerbation of neurological deficits, known as “secondary injury” [36,37]. The secondary lesion is characterized by three consecutive phases: an acute, a subacute, and a chronic phase [29]. The acute phase is characterized by vascular damage, ionic imbalance, free radical production, increased calcium influx, lipid peroxidation, excitotoxicity, inflammation, edema, and necrosis [38]. Additionally, inflammatory cells such as macrophages, T-cells, microglia, and neutrophils infiltrate the site of the lesion, inducing the blood-marrow barrier break-down. These cells release cytokines with pro-inflammatory functions, such as tumor necrosis factor-alpha (TNF-α), interleukin 1-alpha (IL-1α), interleukin 1-beta (IL-1β), and interleukin-6 (IL-6), which reach their peak 6–12 h after injury and remain in circulation for four days [39,40]. These events are followed by a subacute phase where the alteration of ionic homeostasis induces an increase in intracellular calcium, which causes mitochondrial dysfunction, and cell death [41,42]. In particular, the death of oligodendrocytes seems to be responsible for the demyelination of axons [7,43]. Moreover, mitochondrial dysfunction is responsible for the excessive production of reactive oxygen species (ROS), and reactive nitrogen species (RNS) which promote oxidative damage to DNA, oxidation of proteins, and lipid peroxidation that is responsible to aggravate the tissue damage [44]. In the days following the injury, the expansion of the damage leads to the chronic phase that is characterized by the activation of astrocytes that produce excessive levels of the extracellular matrix. These events, together with the hostile environment, favor delayed necrosis and increased neuronal apoptosis. In this way, the processes of cell migration and axonal growth repair are inhibited, inducing cystic cavity formation, axonal death, and maturation of the glial scar (Figure 2) [13,45,46].

After the injury, mechanical compression of the spinal cord results in impaired blood flow followed by ischemia and expansion of the neuronal tissue damage. Therefore, to minimize the damage, early intervention is important, between 8 and 24 h after the damage, through surgical decompression [47]. Surgical decompression improves cord compression, increases vascularity at the injury site, and decreases damage expansion [48]. 

In acute SCI, pharmacological treatment is an important therapeutic strategy that is used to improve neurological function and promote the repair of nervous tissue. Several promising drugs were tested, however, only a few are applicable in SCI patients. All therapeutic strategies that are used are aimed at reducing tissue damage and improving the patient’s quality of life but are not sufficient for neuronal regeneration and for repair interruption of axonal connection in the damaged CNS. Consequently, the repair of the neuroanatomical circuit to resume locomotor function remains an important challenge even today.

## 4. MiRNAs Biogenesis, Structure and Function

MiRNAs are small, single-stranded non-coding endogenous molecules that are 21–25 nucleotides long, characterized by a 5’-phosphate and a 3’-hydroxyl end. MiRNAs negatively regulate gene expression at the post-transcriptional level, binding to the 3’ untranslated region (UTR) or to the 5’UTR region or to the coding region of the target mRNA [49]. MiRNAs perform a “hetero-silencing” mechanism, blocking translation or degrading target mRNA. The same miRNA can aim to different target mRNAs; conversely, different miRNAs can target the same mRNA [50]. The nucleotide sequences of miRNAs are conserved over the course of evolution and their genes can be located both in intergenic regions and within genes of introns or in untranslated exons.

**Figure 3:**
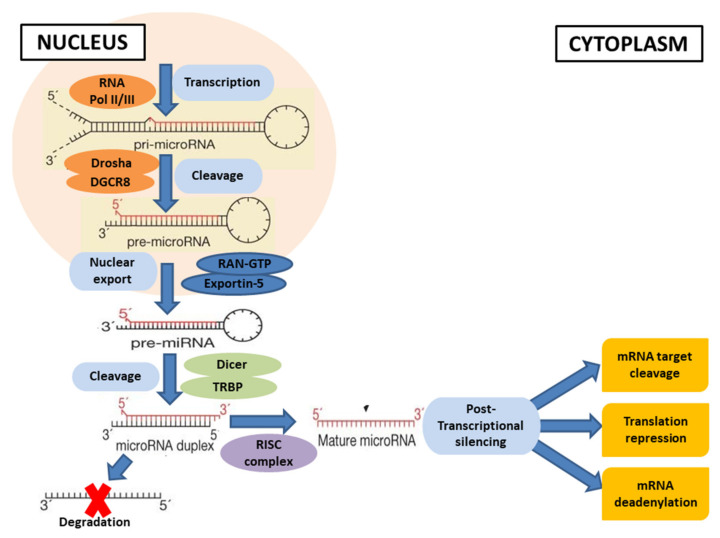
The canonical process of miRNA biogenesis. In this maturation process, miRNA genes are transcribed by RNA polymerase II or III (RNA Pol II/III) to form primary miRNA transcript (pri-miRNA). In the nucleus, this pri-miRNA is cleaved by the microprocessor complex Drosha–DGCR8 to generate pre-miRNA, which is exported by Exportin-5–Ran-GTP from the nucleus to the cytoplasm. In the cytoplasm, pre-miRNA is cleaved by RNase Dicer in a complex with the double-stranded RNA-binding protein TRBP to produce mature miRNA. Subsequently, the functional strand of the mature miRNA is loaded together into the RNA-induced silencing complex (RISC), to perform the post-transcriptional gene silencing, whereas the passenger strand is degraded. Finally, the miRNA induces gene silencing by degradation through translation repression, or messenger RNA (mRNA) target cleavage, or mRNA deadenylation.

The transcription of the genes coding for miRNAs occurs by RNA polymerase II or RNA polymerase III, which gives rise to primary miRNA transcripts, known as pri-miRNAs [51,52]. The two RNA polymerases are regulated differently and recognize specific promoter and terminator elements, thus allowing for different regulatory options [52]. However, it would appear that the pri-miRNAs showed a higher affinity towards RNA polymerase II, an affinity due to the presence in this transcript of typical RNA polymerase II promoters [52]. Subsequently, it is cleaved in the nucleus by a nuclear microprocessor complex that is made up of the RNase III enzyme Drosha (RNASEN) and the protein DGCR8 (DiGeorge critical region 8) [53]. DGCR8 orients the catalytic domain of Drosha allowing the enzyme to cleave the pri-miRNA, obtaining transcripts of about 70 nucleotides that are called pre-miRNA [54]. After nuclear processing, pre-miRNAs are complexed by RAN-GTP proteins and transported from the nucleus to the cytoplasm through Exportin 5 [55,56]. In the cytoplasm, Dicer, an RNase III, promotes pre-miRNA cleavage by generating small RNA of about 22 nucleotides, called miRNAs. A single strand of miRNA is incorporated into a complex called an “RNA-induced silencing complex” (RISC), while the other strand is degraded [57]. The miRNA processing and assembly into the RISC complex are mediated by the RISC loading complex (RLC), consisting of RNase Dicer, Tar RNA binding protein (TRBP), PKR protein activator (PACT), and the main component Argonaute-2 (Aug2) [58,59,60]. The miRNA with the RISC complex binds and regulates specific target mRNAs [57], using two different mechanisms that depend on the complementarity between the miRNA and its target mRNA. The miRNA induces gene silencing by degradation through messenger RNA (mRNA) deadenylation when they are complementary. Conversely, a block of translation occurs when the two sequences own imperfect complementarity [61]. The inhibition of translation can occur either by translational repression during the initial or elongation phase of the mRNA maturation or by a premature detachment of the ribosome (Figure 3) [62].

MiRNAs play an important role in different physiological processes such as cell differentiation and proliferation, immune response, cell death, and metabolism [63,64,65,66]. However, their role in several pathological conditions such as cancer, cardiovascular disease, and diabetes/obesity is also well studied, and their involvement in the development of several neurodegenerative diseases such as Parkinson’s disease, Alzheimer’s disease, and Schizophrenia is also well-known [67]. This is because miRNAs can finely regulate several genes that are involved in the maintenance of neuronal homeostasis [68]. In this regard, it has been shown that the CNS has high concentrations of miRNAs and their gene silencing plays an important role during brain development and in the maintenance of homeostasis of neuronal circuits. Indeed, these molecules are implicated in different processes such as neuron differentiation, neurogenesis, gliogenesis, and synaptic plasticity [69]. Furthermore, the role of miRNAs in neuroinflammation is also widely ascertained. Indeed, they can enhance or inhibit the inflammatory response through their gene silencing [70,71]. Several findings highlight the involvement of miRNAs in the regulation of microglia and the astrocyte activation process and in the control of immune cells such as neutrophils, macrophages, leukocytes, and T- and B-cells [72]. Their role in the differentiation of T and B lymphocytes, in the signaling of Toll-like receptors (TLR) and in the cytokines production has also recently been highlighted [73].

Therefore, given their involvement in several physiological and immune-neuroinflammatory processes, it is important to understand their role after CNS injuries such as SCI.

## 5. MiRNAs Expression in SCI

MiRNAs are known to play important roles in SCI development and plasticity, some of which may be effective therapeutic targets for this pathological condition. Therefore, elucidating the function of miRNAs in SCI may not only further elucidate the SCI pathogenesis but also provide novel therapeutic targets and strategies.

In SCI, the damage is measured by a standard neurological exam, which is challenging and often impossible to perform, especially in the post-acute phases after injury. Therefore, the lack of valid tools to classify the severity of the injury and predict the outcome represents a limitation that hinders the development of therapies for SCI. The identification of a biomarker panel represents an important therapeutic tool for overcoming these obstacles. Several evidences show that miRNAs represent potential biomarkers for the detection and regulation of post-traumatic and neurodegenerative processes [74,75]. Therefore, changes in expression were identified in cerebrospinal fluid (CSF), serum, and plasma of SCI patients and in animal models of SCI [76,77,78,79,80].

A high number of miRNAs are expressed in the mammalian CNS, including the spinal cord, many of which are cell-type specific [18]. For example, it has been observed that miRNA-124 and miRNA-128 are preferentially expressed in neurons, while miRNA-23 is specifically expressed in astrocytes and miRNA-219 in oligodendrocytes [81,82]. They are involved in neural development, neurogenesis, and oligodendrocyte differentiation, therefore, they play an important role in the functions of the spinal cord. For example, miRNA-219 is involved in the differentiation of oligodendrocytes, while miRNA-381, suppressing the expression of Hes1, induces the proliferation of neurocytes [83,84]. This demonstrates how the dysregulation of miRNAs may be implicated in SCI [85].

In this regard, an in silico study evaluated transcriptome expression levels in the spinal cord tissue of rats with SCI at different stages in order to evaluate transcriptome changes before and after injury building a competing endogenous RNA (ceRNA) network. Differentially expressed RNAs were selected, including eight mRNAs, one RGD1564534 long non-coding RNAs (lncRNAs), and miR-29b-5p. The results of the analysis also demonstrated that RGD1564534 can act as a ceRNA and bind to miR-29b-5p by regulating the expression of SCI-related eight mRNAs. Some of these mRNAs are involved in the signaling pathway of hypoxia-inducible factor 1 (HIF-1), a regulator of the response to oxygen supply. HIF-1 protects the nervous system from damage that is induced by ischemic or hypoxic conditions. In addition, during the early stages of SCI, overexpression of HIF-1 can promote vascularization following SCI and favors its recovery. Therefore, miR-29b-5p could play a key role in disease progression by regulating the expression of these eight SCI-related mRNAs and could be a valid biomarker [86].

Several researchers have shown that lncRNAs can regulate mRNAs by binding to miRNAs. The hypothesis was that the expression of specific miRNAs can be decreased due to the ceRNA. A regulatory network of miRNAs that are involved in the physiopathology of many diseases was detected. Wang et al., identified a triple global network by extracting data from the Gene Expression Omnibus (GEO), edited by the National Center for Biotechnology Information (NCBI). Based on the resulting network, target lncRNA-miRNA-mRNA regulatory networks that were associated with SCI were identified and consisted of 13 lncRNA, 93 mRNA, and 9 miRNA, showing their involvement in the pathophysiology of this condition [87].

The expression profile of miRNAs was initially identified in the spinal cord tissue of rats and mice. In the acute phase of SCI, two miRNAs, miR-181a and miR-127, were found to be involved in the regulation of expression levels of cytosolic and secretory phospholipases A2, belonging to the secondary damage event of SCI. There was also an increased expression of miR-221 and miR-1, which target the anti-inflammatory genes annexin A1 and A, and miR-206, miR-152, and miR-214, which instead influence the expression of antioxidant genes [18,88]. In a similar animal model of SCI, a significant increase in miR-146a and miR-129-2 was observed after injury compared to the control in both acute and sub-acute phases [89].

Recently, a high-throughput RNA-seq analysis that was performed on epicenter spinal injury samples that were obtained from three groups with acute traumatic SCI and three shams demonstrated that acute traumatic SCI can dysregulate lncRNA, miRNA, and mRNA expression. Gene ontology (GO) and Kyoto Encyclopedia of Genes and Genomes (KEGG) functional analyses showed that some differentially expressed lncRNAs could play crucial regulatory roles in several mechanisms such as D-glutamine metabolism, D-glutamate metabolism, and receptor binding of high-density lipoprotein particles. The analysis identified miR-21a-5p as the most expressed in the acute traumatic SCI group, identified as the key point of many pathophysiological processes of this condition. The lncRNA/miRNA interaction network has identified lncENSMUST00000195880 as a potential lncRNA that could interact with this miRNA and influence its molecular mechanisms in SCI. Furthermore, miR-28a-3p, miR-1246-5p, miR-135a-5p, and miR-488-3p have also been identified as differentially expressed miRNAs that could bind with lncENSMUST00000195880 and influence the acute traumatic SCI physiopathology [90]. As in the previous study, in the epicenter of spinal cord lesions of a mouse SCI model RNA sequencing analysis identified the first 40 miRNAs that were differentially expressed in the tissues of mice with SCI compared to sham tissues (listed in Table 1). A total of eight of the differentially expressed miRNAs i.e., miR-23a-5p, miR-222-3p, miR-223-3p, miR-22-5p, miR-218-5p, miR-214-5p, miR-21a-3p, and miR-21a-5p, have been selected because of their interactions with ceRNA and mRNA. Therefore, these findings provide new information on identifying new biomarkers and encouraging the development of potential therapies to improve functional recovery during the acute phases of SCI [91].

The study of the expression level of miRNAs in SCI is not limited to the area of damage alone. Indeed, Tigchelaar et al. evaluated the potential of miRNAs as biomarkers of injury severity in a pig (*Sus scrofa*) model of acute traumatic SCI. The results showed an increase in some miRNAs depending on the severity of the disease, most observed 1 and 3 days after the injury. The miRNAs that were differentially expressed in the serum and CSF of the SCI group rather than the control group including miR-133a-5p, miR-378, miR-378b-3p, miR-365-3p, miR-133b, miR- 10b, miR-885-5p, miR-130a, miR-100, miR-208b. Instead, the miR-130a, miR-744, miR-425-5p, miR-130b, miR-423-3p, miR-125b, miR-152, let-7i, miR-100, and miR-30b-5p were differentially expressed in the severe SCI group compared to the moderate SCI group, suggesting that they could be valid biomarkers to distinguish the severity of the disease. Indeed, these miRNAs were differentially expressed in the severe vs. moderate SCI comparison. Finally, miR-486, miR-10b, miR-100, miR-301, miR-378, miR-133a-5p, miR-126-5p, miR-30b-5p, and miR-378b-3p were helpful to distinguishing moderate from mild SCI. These results highlighted how miRNA expression can increase depending on injury severity, representing promising approaches in the diagnosis of disease and in the classification of lesion severity [92]. In light of these data, the same research group, using a new generation sequencing technique, identified the miRNAs that are related to the severity of the disease from the serum and cerebrospinal fluid of patients with acute SCI. Specimens were collected one to five days after injury from patients. The main miRNAs that were identified in the CSF that were related to the ASIA scale after 24 h from the injury were miR-9-5p, miR- 181c-3p, miR-320a, miR-769, miR-9-3p, miR-219-2-3p, miR-432-5p, miR-128-3p and miR-323a-3p. While in serum the main identified miR-192-5p, miR-133a-3p, miR-122-5p, miR-194-5p, miR-4792, miR-1246, miR-208b-3p, miR-499a-5p, and miR-148a-3p [93].

Cai et al., by real-time polymerase chain reaction (RT-qPCR), identified the highly downregulated miR-34a in the serum of patients with traumatic SCI compared to healthy subjects which were higher in SCI patients with remission than in the non-remission group. This miRNA can also be used in the monitoring of the neurological remission process in SCI conditions [94].

The next-generation sequencing allowed the analysis of the profiles of miRNAs that were contained in the serum exosomes of rats with acute SCI. Unlike free exosomes, exosomal miRNAs have the advantage of being contained in structures whose membrane can improve their stability and allow them to pass through the blood-brain barrier. In the study that was performed by Ding et al., rats with acute SCI showed deregulation in serum exosomal miRNAs that were associated with the lesion compared to sham rats. In detail, 10 upregulated miRNAs were detected; conversely, 10 were downregulated following the lesions (as can be seen in Table 1). The KEGG enrichment assays revealed as targets of miRNAs differentially expressed some enriched pathways that are involved in SCI such as the Wnt signaling pathway, long-term potentiation, axon guidance, ECM-receptor interaction, and focal adhesion. Of note, among these deregulated miRNAs, miR-125b-5p, miR-152-3p, and miR-130a-3p may be the most specific and readily identifiable diagnostic markers in acute SCI [95]. 

Subsequently, the same research group also evaluated the transcriptomic profile of exosomal serum miRNAs of rats with SCI in the subacute phase. In this phase, the 10 most upregulated and 10 downregulated serum exosome miRNAs were also found seven days after the injury (as can be seen in Table 1). Additionally, all miRNAs that were deregulated in SCI rats compared to the sham group were further compared with miRNAs previously found in the spinal cord or biological fluids after injury. Among 16 miRNAs already found in previous studies, miR-672 and miR-15b were downregulated; while the miR-485, miR-30b, miR-26b, miR-23b, miR-223, miR-21, miR -211, miR-200c, miR-195, miR-17, miR-133a, miR-125b-1, miR-124, and miR-103 were upregulated. The pathways that were targeted by the identified miRNAs included Mitogen-activated protein kinase (MAPK) signaling pathways, Ras-associated protein-1 (Rap1) signaling pathway, cyclic guanosine monophosphate (cGMP)-protein kinase G (PKG) signaling pathways, Wnt signaling pathways, metabolic pathways, axonal guidance, endocytosis, and necroptosis. As these biological processes and pathways are involved in SCI physiopathology, these evidence highlights the involvement of miRNAs in SCI and their importance as biomarkers in this pathology [96].

In a study that was performed by Ye et al., the differential expression of miRNAs in the plasma of healthy subjects was evaluated compared with subjects with acute or chronic SCI. The analysis identified 383 miRNAs that were differentially expressed in patients with acute or chronic SCI compared to healthy subjects. It was also observed that 71 miRNAs were differentially expressed in SCI subjects with neuropathic pain compared to patients without neuropathic pain. Among these, hsa-miR-19a-3p and hsa-miR-19b-3p were selected based on the *p*-value. The results showed that the levels of both miRNAs were higher in SCI patients than in healthy subjects, and in turn, the expression levels were higher in patients with acute SCI than in those with chronic SCI. Furthermore, it was also observed that both hsa-miR-19a-3p and hsa-miR-19b-3p have a discriminatory capacity between subjects with SCI without neuropathic pain and subjects with SCI without pain. The common targets that were shared by hsa-miR-19a-3p and hsa-miR-19b-3p were identified using miRTarBase, and included Phosphatase and Tensin homolog (PTEN), potassium channel subunit voltage-gated, RUNX family transcription factor 3 (RUNX3), tumor protein p53 (p53), and Rap guanine nucleotide exchange factor (2Rapgef2). MiR19a-targeted Methyl-CpG Binding Protein 2 (MeCP2), Ras homolog family member B (RhoB), suppressor of cytokine signaling 1 (SOCS1), Leucine-rich repeats and Immunoglobulin-like domains 1 (LRIG1), and Peroxisome Proliferator-Activated Receptor Alpha (PPARα); while the miR-19b targeted signal transduction and activator of transcription 3 (STAT3) and FMR1 Autosomal Homolog 1 (FXR1). Neuropathic pain occurs at or below the lesion level, and it is refractory to most treatments, thus the identification of biomarkers could be useful in identifying new pharmacological and non-pharmacological strategies [97].

**Figure 4:**
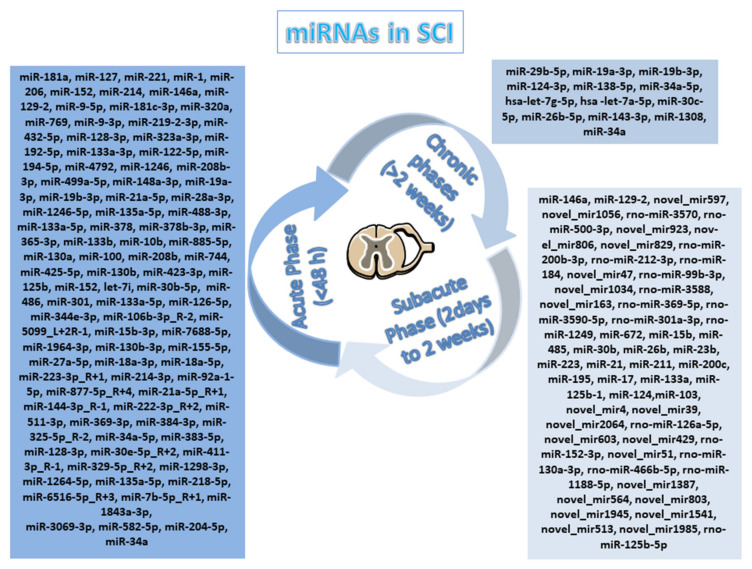
Schematic representation of miRNAs in different phases of SCI. The figure illustrates the miRNAs deregulate after SCI in acute, sub-acute, and chronic phases, which could be implicated as biomarkers in SCI animal models and SCI patients. The image was created using the image bank of Servier Medical Art (Available online: http://smart.servier.com/, accessed on 7 June 2022), licensed under a Creative Commons Attribution 3.0 Unported License (Available online: https://creativecommons.org/licenses/by/3.0/, accessed on 7 June 2022).

The data that are illustrated in the aforementioned papers, summarized in Table 1, show that changes in miRNAs expression, both locally and systemically, can be involved in the pathogenesis of SCI making them potential dynamic biomarkers across the different stages and in the severity grade of the lesion (Figure 4).

## 6. MiRNAs for Neuronal Regeneration in SCI

MiRNAs are important regulators of the genome, therefore, they can be implicated in different mechanisms of action against CNS lesions, such as SCI. It has been observed that part of the neuronal and axonal regeneration processes in CNS lesions that are caused by stroke, cranial trauma, and SCI, occurs in different areas of the brain such as the subventricular zone (SVZ) or the subgranular layer (SGL) of the dentate gyrus in the hippocampus and can be modulated in part by miRNAs [98,99].

In an animal model of SCI, next-generation sequencing in the sensorimotor cortex of layer V was performed to assess miRNA dysregulation 12 h and 3 days after SCI. MiR-7b-3p was the only significantly upregulated miRNA. Targets Functional Analysis highlighted WAS/WASL interacting protein family member 2 (*Wipf2)*, a gene that is implicated in neurite extension, as a target of this miRNA. Furthermore, 16 genes that were involved in neuronal regeneration were downregulated in the animal cortex after injury and were identified as potential targets of miRNAs miR-7b-3p, suggesting its possible involvement in SCI-related gene regulation. Transfection of miR-7b-3p mimic in Neuro2a (N2a) cells confirmed the *Wipf2* gene as its direct target, reducing its expression after transfection. Therefore, the in vitro study showed that the miRNAs, despite not stimulating neurite growth, nevertheless kept the cells in a more plastic stage and protected the primary cortical neurons from the apoptotic cascade that is activated after SCI [100].

Another promising miRNA that is involved in regeneration after SCI is miRNA-124. Rats with SCI were treated with agomiR-124 for 14 days. AgomiR-124 improved neuronal loss and reduced astrocyte activation, while simultaneously increasing neurofilament-200 (NF-200) expression in the dorsal horn. GO and KEGG analysis identified the *tal1* gene as a potential target of miR-124. MiR-124 mimic reduced the expression of the neuronal-nuclei (NeuN) level, demonstrating that it suppresses the differentiation of SH-SY5Y into neuron-like cells but enhances their proliferation. Thus, the miR-124/tal1 axis can be used as a possible treatment for repopulating the neural stem cells after SCI [101]. The therapeutic efficacy of miR-124 was also confirmed in a study that was performed by Cui et al. in mouse spinal cord progenitor cells (SC-NPCs). The overexpression of miR-124 activated Wnt/β-catenin signaling, which in turn was mediated by Neat1 upregulation during SC-NPCs differentiation. In vitro, miR-124 and Neat1 protected SC-NPCs from apoptosis and improved their migratory capacity. Additionally, miR-124 induced neurite length both in vitro and in vivo. Furthermore, in SC-NPCs, both the transfection with miR-124 and Neat1 increased the expression of some markers of neuronal differentiation such as Tuj1 (class III beta-tubulin) and microtubule-associated protein 2 (Map2); contrarily, they reduced the expression of glial fibrillary acidic protein (GFAP). Even in vivo, the percentage of GFAP-positive in the glial scars was decreased in SCI animals that were treated with miR-124. In vivo, 1 or 2 weeks after injection, miR-124 improved motor skills and promoted functional recovery in SCI animals [102]. 

More than conventional therapy, cell transplantation is also a therapy that is used for promoting the repair of spinal cord tissues after SCI. In this context, Song et al. evaluated the effect of miRNA-124 contained in the recombinant lentiviral vector (miR-124-LV) and transfected into bone marrow mesenchymal stem cells (BM-MSCs). The luciferase test identified the pyridoxal kinase (PDXK) gene as a target of miR-124. The overexpression of miR-124 and the silencing of the PDXK gene increased the ability of BM-MSCs to repair the lesion of animals with SCI. The lesioned animals that were transplanted with miR-124-LV transfected BM-MSCs significantly increased the level of protein expression related to repair SCI, such as thyrotropin-releasing hormone (TRH), prostaglandin I2 (PGI2), and gangliosides. Moreover, 7 and 24 days after transplantation with miR-124-LV transfected BM-MSCs improved blood-brain barrier (BBB) scores compared to animals with untreated SCI and also compared to the control group, demonstrating the role of miR-124 in enhancing the ability of BM-MSCs to repair injured tissues [103].

Activation of the Phosphoinositide 3-kinase (PI3K)/protein-kinase B (AKT) pathway, inhibited spinal cord apoptosis and improved motor function after SCI; therefore several miRNAs target the effectors of this pathway to promote neuronal survival, and differentiation and protect cells from apoptosis. In this regard, it was observed that miR-448 is upregulated following SCI and its expression gradually increases with the progression of the disease. The double luciferase reporter gene test demonstrated that miR-448 targets the Bcl-2 gene, suggesting the potential role of this miRNA in regulating neuronal apoptosis. Treatment with the miR-448 inhibitor in the spinal cord of mice with SCI promoted the increase of p-PI3K, p-AKT, and Caspase 3 (Casp3) expression. This result demonstrated that miR-448 protected neurons from neuronal apoptosis, activating the PI3K/AKT pathway, and it promoted the regeneration of motor neurons. Furthermore, inhibition of the miR-448 inhibitor improved motor function of the upper limbs by improving the grip of mice with SCI. Treatment with Bcl-2 siRNA obviously reduced the expression of Bcl-2 at both mRNA and protein levels, reversing the regenerative effects of the miR-488 inhibitor. These data confirmed the involvement of miR-488 in promoting motor recovery after SCI by modulating the PI3K/AKT/Bcl-2 axis [104].

Noteworthy, the miRNAs interaction and the PI3K/AKT signalling pathway can also regulate astrogliosis and astrocyte proliferation. Since astrocytes are key players in the glial scar generation process that inhibit repair after SCI, understanding the underlying mechanisms could be useful for developing therapeutic strategies. To evaluate the role of miR-140, human astrocytes that were exposed to lipopolysaccharide were transfected with miR-140 imitators or miR-140 inhibitors. The overexpression of miR-140 resulted in reduced viability and cell proliferation. Furthermore, it reduced the expression of brain-derived growth factor (BDNF), a regulator of astrocyte proliferation and differentiation, confirmed as a target of this miRNA. Furthermore, miR-140 also decreased protein levels of p-PI3K and p-AKT, demonstrating that the regulatory effect of miR-140 and BDNF on astrocyte proliferation was mediated by the PI3K/AKT pathway. Conversely, transfection of astrocytes with the miR-140 inhibitor increased cell viability and proliferation and increased BDNF levels [105]. In the same model, the miR-211 inhibitor increased its proliferation and protein levels of BDNF, p-PI3K, and p-AKT. Therefore, these studies demonstrated that miR-140/BDNF and miR-211/BDNF could serve as promising targets for counteracting the reactive proliferation of astrocytes after SCI [106]. 

Another important therapeutic target that is implicated in scar formation and functional recovery is miR-21, which appears to be involved in the regulation of the transforming growth factor-β (TGF-β)/Small mother against decapentaplegic (SMAD) signaling pathway, and the PTEN/PI3K/AKT signaling pathway [18,107]. In a study that was performed by Xie et al., the therapeutic effect of miR-21 was investigated in a model of acute thoracic SCI. After 14 days following the surgery, the group that was treated with the miR-21 knockdown vectors showed an improvement in the Basso Mouse Scale (BMS) scores; while the morphological analysis showed an improvement in the injured tissues, the morphology of the neurons improvement, and signs of axonal regeneration. Furthermore, miR-21 knockdown induced a decrease in the inflammatory response, with a consequent reduction in serum levels of TGF-β1, TNF-α, and IL-1β and reduced activity of phosphorylation of AKT. In this way, miRNA-21 inhibition can be employed as a therapeutic strategy to induce motor recovery and reduce the inflammatory response. In addition, miR-21 inhibition also increased BDNF expression, further highlighting its important role in axonal and neuronal regeneration [108]. The role of miR-21-5p in regulating the formation of fibrotic scars has also been demonstrated in a mouse model of contusion injury. In lesion tissues, miR-21-5p was found to be upregulated. This mechanism could be a potential role of miR-21-5p in enhancing the pro-fibrogenic effect of TGF-β1 in spinal fibroblasts. Conversely, miR-21-5p knockdown protected the tissue from these mechanisms and also promoted functional motor recovery after SCI [109]. In primary spinal fibroblasts after 48h post-scratch, microarray analysis and qRT-PCR identified increased expression of miR-21a-5p. To investigate the role of miR-21a-5p, mouse spinal fibroblasts were transfected with miR-21a-5p mimics/inhibitors. The overexpression of miR-21a-5p in spinal fibroblasts that were subjected to scratch damage promoted an increase in fibrogenic activity. Moreover, it was observed that miR-21a-5p mimics triggered a significant increase in Ki-67 and B-cell lymphoma (Bcl-2) protein expression and decreased level of Bcl-2-associated X protein (Bax), thus showing that this miRNA increased proliferation and attenuated apoptosis of spinal fibrosis after mechanical trauma. Furthermore, the bioinformatic analysis identified *SMAD7* as a target of miR-21a-5p. In compliance with this data, Western blot analysis showed that miR-21a-5p overexpression significantly reduced SMAD7 in the scratched spinal fibroblast model, but on the contrary, increased SMAD2/3 phosphorylation.Therefore, miR-21a-5p-induced SMAD2/3 phosphorylation activated the TGF-β/SMAD signaling pathway, emphasizing its role in promoting spinal fibrosis after mechanical trauma [110].

Interestingly, changes in miRNA expression after SCI could alter the growth potential of damaged neurons. Rat dorsal root ganglions (DRGs) extend axonal projections in culture in the absence of exogenous growth factors and can rapidly extend axons through translational regulation of mRNAs. In compliance with this evidence, it was noted that miR-21 was increased also in adult DRG neurons after axotomy; conversely, the level of miR-199a-3p was reduced. After transfection into naive adult DRG cultures with biotinylated miR-21 and miR-199a-3p mimics, it was observed that the miR-21 overexpression promoted neuritic growth, on the contrary miR-199a-3p reduced growth and also protein synthesis. Furthermore, miR-21 stimulated axonal growth and decreased PTEN protein, reducing protein synthesis, while miR-199a-3p attenuated its growth by reducing the mechanistic target of rapamycin (mTOR) protein and consequently decreasing neuronal protein synthesis. Thus, the overexpression of the two miRNAs are involved in axon growth regulation through the regulation of the PTEN/mTOR pathway [111]. mTOR is also a potential target of the miR-99b-5p. Indeed, quantitative RT-qPCR showed that it was overexpressed in the bone marrow of SCI mice. Subsequently, neurons that were transfected with miR-99b-5p inhibitor protected neurons from SCI-induced apoptosis. In addition, the transfection also promoted neurite growth which was reduced in the SCI group and increased mTOR levels [112].

However, miR-29a also regulates the PTEN/mTOR signalling pathway by targeting PTEN, reducing its expression and promoting neurite growth. To confirm this mechanism, mice were subjected to injections into the lesion site with a recombinant lentiviral vector to promote miR-29a expression. Overexpression of miR-29a significantly reduced PTEN expression, increased phosphorylation of AKT and S6, two proteins of the PI3K/AKT/mTOR signalling pathway, and expression of the NF-200 protein, a key marker that is associated with axonal regeneration. Axonal regeneration that was mediated by treatment with the recombinant lentiviral vector-miR-29a was also demonstrated by quantitative imaging analysis. Moreover, the recombinant lentiviral vector promoted motor functional recovery of the hind limbs demonstrating the importance of these miRNAs as a regulator for axon regeneration and as a possible therapeutic target for SCI recovery [113]. Consistent with these data, in an in vivo model of SCI, the expression of miR-29b was downregulated. Mice that were treated with miR-29b lenti-virus vector induced an improvement in the lesion, reducing neuronal loss and cavity number, and inducing neuronal survival in the gray matter ventral horn. In addition, the overexpression of miRNAs protected neurons from SCI-induced apoptosis decreasing Bax expression and improving Bcl-2 expression [114]. MiR-29b that was delivered within exosomes that were extracted from BM-MSCs also showed protective roles on SCI. SCI rats were injected intravenously with miRNA-29b exosomes and with miR-29b-modified BM-MSCs. Treatment with miRNA-29b-exosomes and miR-29b-BM-MSCs induced an increase in miR-29b expression in the spinal cord of SCI rats. MiR-29b overexpression ameliorated SCI clinical signs and motor function, improving the Basso Beattie Bresnahan score. Moreover, it was observed that treatment with miRNA-29b-exosomes and miR-29b-BM-MSCs induced neuronal regeneration, enhancing the number of NF-200 and growth-associated protein 43 (GAP-43) positive neurons, and decreasing the number of contractile nerve cells and glial fibrillary acidic protein (GFAP) positive neurons, with a significant improvement in the miRNA-29b-exosomes group than in the miRNA-29b BM-MSCs group. This demonstrated that exosomes are promising therapeutic agents and their manipulation with miRNAs makes them even more encouraging [115].

Apoptosis has been identified as a vital mechanism of cell death in many neurological diseases and in SCI. Hence, genes that are related to apoptosis can become potential targets of miRNAs. With regard to that, Huang et al. evaluated the effect of miR-494 on neuronal apoptosis in the damaged area and tested its repair effect on SCI rats. Exosomes that were modified with miR-494 were co-cultured with the DRG and rat NR8383 macrophages of spinal cord injury. In vitro, a reduction of Casp3 and Bax and an increase of the anti-apoptotic protein Bcl-2 in the DRG of the exosomes that were modified with the miR-494 group has been reported. MiR-494-modified exosomes reduced pro-inflammatory cytokines such as TNF-6 and IL-1α and resulted in increased the expression of anti-inflammatory cytokines IL-8 and IL-10, related to the M2 polarization of rat NR8383 macrophages. Also in vivo, miR-494-modified exosomes downregulated GFAP expression and promoted an increase in neurofilament in SCI rats, and improved motor function [116]. 

Plasticity in the damaged spinal cord is modulated in part through miRNAs that regulate mTOR signalling and may indicate an increase in the regenerative potential of SCI-affected neurons. Indeed, the enriched GO terms and KEGG pathways demonstrated that miR-26a has several target genes that are involved in multiple biological pathways including PTEN and mTOR pathways. Transfection of PC12 cells with miR-26a-modified MSC-derived exosomes reduced PTEN expression and PI3K, AKT, and mTOR protein phosphorylation was increased to promote neurofilament generation in PC12 cells and nerve regeneration. The miR-26a exosomes were injected into the tail vein immediately after SCI, promoting functional recovery in rats, and inducing neuronal and axonal regeneration by targeting the PTEN and mTOR pathways. Furthermore, modulation of this pathway also has attenuated excessive autophagy, thus promoting regeneration after SCI [117]. Also, miR-133b-modified adipose tissue-derived MSCs (AT-MSCs)-secreted exosomes showed their protective role in the recovery of neurological function after SCI. After transfection, the exosomes that were secreted by AT-MSCs showed a high expression of miR-133b. MiR-133b mimics overexpressed the level of miR-133b in SCI animals compared to the control group, conversely, downregulating the expression of Ras homolog gene family member A (RhoA), promoting the recovery of neurological function in mice with SCI. It was also observed that exosomes that were modified with miR-133b significantly increased levels of NF-200, GAP43, GFAP, and myelin basic protein (MBP), thus affecting the regeneration-related signalling pathway of the axons [118]. Previously, Teis et al. demonstrated that miR-133b promoted growth in vitro. While in vivo, lentivirus-encoding miR-133b induced functional recovery in mice with SCI four weeks after injury and virus injection. This improvement could be related to the downregulation of expression levels of the RhoA. RhoA is known to inhibit neurite growth, therefore, these data encourage the application of this miRNA in SCI therapy [119]. In compliance with these studies, treatment with miR-133b exosomes was shown to promote the functional recovery of the upper limbs in an in vivo SCI model. Additionally, miR-133b exosome treatment also reduced lesion volume, protected the neurons from death, and improved axon regeneration. Treatment with miR-133b exosomes increased the expression levels of the NF-200 and GAP43 proteins while reducing RhoA expression, which were consistent with previous studies. In the damaged spinal cord, the treatment also increased extracellular regulated kinase 1/2 (ERK1/2) protein phosphorylation, and cAMP response element-binding protein (CREB) and STAT3 proteins phosphorylation, two transcription factors that are involved in neurite growth [120]. Treatment with exosomes that were secreted by MSCs that were modified with miRNAs can be a valid approach for SCI management.

The role of miRNAs in axonal growth during differentiation and injury is a new area of research. In this regard, it was observed that also miR-20a can promote the growth of cortical neurons. Its expression has been shown to decrease in a time-dependent manner following a spinal cord dorsal column lesion. DRG neurons that were transfected with miR-20a mimics showed significantly longer neurites compared to the control group. The transfection also increased GAP43 expression, contrarily, it reduced the levels of RhoA and PSD-95/Dlg/ZO-1-Rho guanine nucleotide exchange factor (PDZ-RhoGEF). The same result was obtained in vivo four weeks after recombinant adenovirus of miR-20a administration. The results demonstrated that the upregulation of miR-20a in DRG neurons and the dorsal column promoted neurofilament synthesis, neurite regeneration, and sensory conduction function recovery targeting the PDZ-RhoGE/RhoA/GAP43 axis. This miRNA could be used as a novel strategy for promoting the sensory function recovery post-SCI [121]. 

Together with miR-20a, miR-30b restores the sensory conductive function of the spinal cord. MiR-30b also induces axon growth in sensory neurons after transfection with miR-30b agomir. Bioinformatic analysis predicted that miR-30b could target Semaphorin 3A (Sema3A) and induce its degradation in primary sensory neurons. Since Sema3A is a ligand of the co-receptor PlexinA1 and Neuropilin 1 (NRP-1), transfection of cells with miR-30b agomir was observed to reduce the formation of Sema3A-NRP-1 and Sema3A-PlexinA1 complexes, demonstrating that miR- 30b regulates the growth of axons by degrading Sema3A. Furthermore, transfection with miR-30b agomir was shown to reduce the expression of GTP-RhoA and Rho-associated protein kinase (ROCK), therefore, this could be the downstream mechanism of Sema3A to regulate neurite growth. In vivo treatment with miR-30b agomir, following the results of the in vitro study, demonstrated that miR-30b agomir promoted axon growth by regulating the Sema3A/PlexinA1-NRP-1/RhoA pathway/ROCK. This also improved sensory conductive function after spinal cord dorsal column lesion [122].

Also, the miR-200b-3p targeted RhoA. Indeed, it was observed that miR-200b-3p mimics have decreased the RhoA and Rock1 gene expression in neural stem cells. Moreover, it was observed that miR-200b-3p was critical to promoting neuronal differentiation that was induced by Wnt family member 5A (Wnt5a), ligand for members of the frizzled family of seven transmembrane receptors. This result was also confirmed in vivo, where miR-200b-3p played a key role in promoting the differentiation of transplanted neuronal stem cells by inhibiting the RhoA pathway, inducing lesion repair and functional recovery in SCI mice [123]. Other miR-200 family members’ deregulation results in the loss of neural progenitor identity and promote neuronal or glial differentiation. Indeed, miR-200a has been identified as an important player in spinal cord regeneration in axolotls, a model of regeneration. In this study, monitoring of stem cells in the spinal cord of axolotls showed that miR-200a, after injury, binds to the mesodermal marker brachyury and it represses the fate of mesodermal cells. When a spinal cord injury occurs, the miR-200a levels remain elevated, which inhibits β-catenin levels, potentially stabilizing a neural stem cell identity in cells that are adjacent to the injury site. In this way, after the injury, miR-200a stimulates the stem cells that are present in the spinal cord of the axolotls to differentiate into neurons and glial cells [124].

Cognitive deficits and mental disorders may occur following SCI. Therefore, restoring sensory function could improve the quality of life of SCI patients. In this context, primary sensory neuron cultures were taken from DRG of adult Wistar rats. The addition of epidermal growth factor (EGF) increased neurite growth after 30 min and one hour after exposure compared to controls. While the level of phosphorylated-epidermal growth factor receptor (p-EGFR) increased in primary sensory neurons after 30 min of application, conversely the level decreased after one hour. Therefore, prolongation of EGF treatment did not further increase neurite elongation. This poor regenerative capacity could be due to Casitas B-lineage lymphoma (CBL) which is involved in the degradation of phosphorylated EGFR. Therefore, cultured primary sensory neurons were transfected with CBL small interfering RNA (siRNA) to downregulate CBL expression. Downregulation raised the levels of phosphorylated EGFR. To demonstrate the involvement of miRNAs in neurite growth, bioinformatic analysis allowed to identify miRNA-22-3p, a neuron-specific miRNA [125], as a potential miRNA targeting CBL. The overexpression of miR-22-3p reduced the level of CBL and increased the p-EGFR, demonstrating that this miRNA could increase neurite growth by eliminating the inhibitory effect of CBL on p-EGFR. Furthermore, miR-22-3p mimic was observed to raise the levels of phosphor-STAT3 (p-STAT3) and phosphor-growth-associated protein 43 (p-GAP-43). Also in vivo, miR-22-3p could improve EGFR signaling and restore spinal cord sensory conductive function through CBL/p-EGFR/p-STAT3/GAP43/p-GAP43 axis regulation [126].

MiR-615 is another potential therapeutic target that is involved in neuron survival and axonal regeneration. It was observed that induced neural differentiation in neuronal stem cells directly targets LRR and Ig domain-containing NOGO receptor-interacting protein 1 (LINGO-1), a potent negative regulator in neuron survival and axonal regeneration. Indeed, LINGO-1 inhibition by miR-615 may contribute to neuronal differentiation with the short process via LINGO-1/RhoA or EGFR signal pathways. Also in vivo, the intrathecal administration of miR-615 agomir in SCI rats inhibited the translation of LINGO-1, increased neuronal survival, enhanced axonal extension and myelination, and improved recovery of hindlimbs motor functions [127].

MiR-125b could be involved in the regeneration process. In a mouse model of cervical SCI, in the injury area it was observed a significant reduction of miR-125b. Janus Kinase 1 (*JAK1)* and *STAT1* were predicted as the target genes. Indeed, a significant increase was observed in the mRNA levels of p-JAK1 and p-STAT1 in the miR-125b group than those in the SCI group. The JAK/STAT pathway is involved in cell growth, differentiation, and survival. Therefore, by targeting this pathway, the overexpression of miRNAs could induce regeneration and repair processes. Indeed, it was observed that the overexpression of miR-125b promoted an increase in axons in the lesion area and induced neuronal protective effects by reducing apoptosis and inflammatory responses in neurons. Additionally, after seven days of injury, the mice improved front leg function and grip strength [128]. 

Emerging evidence has shown that cell death in the spinal cord after injury can also be caused by an inflammatory environment and increased synthesis and release of IL-1β [129]. In one study, human neural stem cells were treated in vitro with IL-1β and expression of kinesin family member 3B (KIF3B) and nitric oxide synthase interacting protein (NOSIP), two important modulators of neural cell response and repair during SCI, were evaluated. It was seen that IL-1β increases the expression of miR-372 that has inhibitory activity towards KIF3B and NOSIP through the activation of nuclear factor-kappa B (NF-кB). Therefore, the attenuation of the expressions of these two genes can worsen SCI and hinder the axonal regeneration that is required for functional recovery. Treatment with anti-miR-372 resulted in the blocking of gene repression of KIF3B and NOSIP. The inhibition of miR-372 improved the recovery of SCI rat function and saved the reduction of post-injury motor activity that was induced by IL-1β [130]. 

MiR-210 also has a neuroprotective effect by suppressing neuronal apoptosis and regulating inflammatory reactions in rats after acute SCI. An adeno-associated virus was used to mediate the overexpression of miR-210 and was injected into the spinal cord. There was a reduction in Casp3 and -8 that resulted in a reduction in apoptotic events. In addition, adeno-associated virus-miR-210 resulted in an anti-inflammatory response through the reduction of inflammatory cytokines IL-1β and TNF-α and an increase in IL-10. The neuroprotective effect was determined by the inhibition of protein tyrosine phosphatase 1B (PTP1B), resulting in increased vascular endothelial growth factor A (VEGF-A) and the activation of PI3K/AKT signaling. These results indicate that adeno-associated virus-miR-210 may be a therapeutic strategy for treating acute SCI [131].

**Figure 5:**
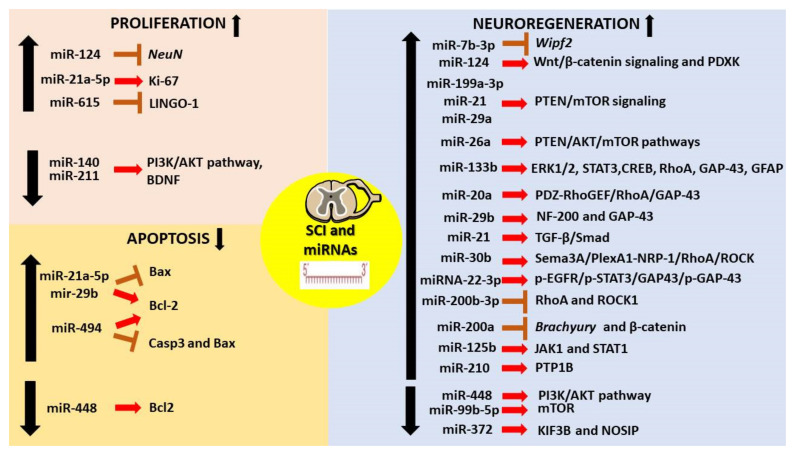
Summary of the roles of essential miRNAs in SCI. SCI affects the expression of miRNAs, which are known to regulate processes such as cell proliferation and apoptosis. Particularly, miRNAs are essential for neuroprotection as it induces neuroregenerative functions by targeting and downregulating several molecular pathways. The image was created using the image bank of Servier Medical Art (Available online: http://smart.servier.com/, accessed on 7 June 2022), licensed under a Creative Commons Attribution 3.0 Unported License (Available online: https://creativecommons.org/licenses/by/3.0/, accessed on 7 June 2022). Neuronal-Nuclei: NeuN; Neurofilament-200: NF-200.

Table 2 highlights the different mechanisms by which miRNAs exert their function after SCI. In particular, miRNAs have been observed to regulate processes such as proliferation and apoptosis and show neuroregenerative effects by targeting different molecular pathways (Figure 5).

## 7. Challenges, Perspectives and Future Objective

The inhibitory microenvironment of the spinal cord after injury reduces the recovery of the neurological deficit, also modifying the expression of miRNAs. These changes have been shown to induce effects on apoptosis, astrogliosis, oligodendrocyte development, and neuronal regeneration, demonstrating their involvement in events that are related to spinal damage. In this context, it was observed that in neurons whose axon has been injured, some miRNAs seem to support the survival of injured neurons and maintain conditions of greater plasticity, which can make them more ready to trigger regenerative phenomena. Therefore, miRNA-based molecules may be a viable therapeutic alternative after SCI. However, a major challenge in miRNA-based therapies is to avoid degradation by circulating RNAases or in the endocytic compartment of cells. Although chemical modifications have been made to overcome this problem, the half-life of some constructs can be short and require repeated injections or infusions. Another problem in the therapeutic field of miRNAs is caused by the potential off-target effects. Indeed, some imitations of miRNAs can cause off-target effects, due to the ability of miRNAs to interact with other genes, inducing unwanted side effects or even activating pathways that counteract the protective effects. Therefore, techniques are needed to increase the specificity of the effects of miRNAs on selected target genes to block off-target effects. Similar to all pharmacological agents, the administration of miRNAs for CNS lesions is complicated by the need to penetrate the BBB. This explains why most studies employ administration via intrathecal or subarachnoid injections. It would appear that the use of exosomes, with or without stem cells, or miRNA-based engineered nanoparticle drugs can improve the ability to cross the BBB and avoid delivery to unwanted sites.

Most of the studies evaluating the influence of miRNAs on SCI are conducted in animals and this makes application to humans difficult, so further investigations are needed to allow translation to the clinic. Despite these limitations, we believe this review can help better understand the regulatory mechanisms of miRNAs following SCI and can encourage future experimental studies to get closer to finding a treatment for SCI.

## 8. Conclusions

Several and recent preclinical evidence highlights the role of miRNAs as useful therapeutic tools that modulate molecular processes and improve functional recovery in SCI. In this review, we have summarized some studies that highlight how miRNA expression triggers complex interactions and changes at the cellular and protein levels that influence the pathophysiology of SCI. The manipulation of miRNA expression may provide an opportunity to develop improved therapeutic and clinical interventions to address the consequences of SCI since they promote axonal growth, cell regeneration, neuroplasticity, and facilitates functional recovery. The ability of miRNAs to modulate gene expression makes them valid candidates as therapeutics for neurogenesis after SCI. Collectively, the available results from preclinical studies suggest that targeting miRNA dysregulation is an attractive therapeutic strategy for this condition. However, the current understanding of the mechanisms underlying SCI pathogenesis is still limited and further miRNAs that are implicated in this pathological process remain to be discovered. Therefore, further studies are needed to understand the functions and objectives of SCI-related miRNAs. Moreover, despite the great focus on miRNAs as possible therapeutic tools, there is a lack of miRNA mimic studies for SCI patients, so clinical trials are needed to evaluate the therapeutic implications of miRNAs.

## Figures and Tables

**Table 1:** miRNA expression in the spinal cord tissue, cerebrospinal fluid, serum, and plasma at different periods post-injury.

miRNAs	Period Post-SCI	Sample	Targets	Potential Effects	Type of Study	Ref.
miR-29b-5p	SCI	-	*IFNGR1, STAT2, CYBB, NFATC1, FCGR2B, HMOX1, TLR4, and HK2*	miR-29b-5p, regulating mRNAs SCI-related, could play a key role in disease progression and could be a valid SCI biomarker	In silico	[86]
miR-124-3p, miR-138-5p, miR-34a-5p, has-let-7g-5hashsa -let-7a-5p, miR-30c-5p, miR-26b-5p, miR-143-3p, miR-1308	SCI	-	-	-	In silico	[87]
miR-21a-5p, miR-28a-3p, miR-1246-5p, miR-135a-5p and miR-488-3p	Acute Traumatic SCI	Lesion epicenter of spinal tissues	lncENSMUST00000195880	These miRNAs were identified as differentially expressed miRNAs that could bind with lncENSMUST00000195880, in this way could play crucial regulatory roles in many pathophysiological processes of SCI such as D-glutamine metabolism, D-glutamate metabolism, and receptor binding of high-density lipoprotein particles	In vivo (mouse)	[90]
miR-344e-3p, miR-106b-3p_R-2, miR-5099_L+2R-1, miR-15b-3p, miR-7688-5p, miR-1964-3p, miR-130b-3p, miR-155-5p, miR-27a-5p, miR-18a-3p, miR-18a-5p, miR-223-3p_R+1, miR-214-3p, miR-92a-1-5p, miR-28a-3p, miR-877-5p_R+4, miR-21a-5p_R+1, miR-144-3p_R-1, miR-222-3p_R+2, miR-511-3p, miR-369-3p, miR-384-3p, miR-325-5p_R-2, miR-34a-5p, miR-383-5p, miR-128-3p, miR-30e-5p_R+2, miR-411-3p_R-1, miR-329-5p_R+2, miR-1298-3p, miR-1264-5p, miR-135a-5p, miR-218-5p, miR-6516-5p_R+3, miR-488-3p, miR-7b-5p_R+1, miR-1843a-3p, miR-3069-3p, miR-582-5p, miR-204-5p	Acute Traumatic SCI	Lesion epicenter of spinal tissues	-	These significant changes in miRNA expression are candidates as biomarkers in acute SCI	In vivo (mouse)	[91]
miR-133a-5p, miR-378, miR-378b-3p, miR-365-3p, miR-133b, miR-10b, miR-885-5p, miR-130a, miR-100, miR-208b, miR-744, miR-425-5p, miR-130b, miR-423-3p, miR-125b, miR-152, let-7i, and miR-30b-5p, miR-486, miR-301, miR-133a-5p, miR-126-5p	Acute Traumatic SCI	CSF and serum	-	These significant changes in miRNA expression are candidates as biomarkers of injury severity after SCI.	In vivo (pig)	[92]
miR-9-5p, miR-181c-3p, miR-320a, miR-769, miR-9-3p, miR-219-2-3p, miR-432-5p, miR-128-3p and miR-323a-3p	Acute Traumatic SCI	CSF	-	These significant changes in miRNA expression are candidates as biomarkers of injury severity after SCI.	In human	[93]
miR-192-5p, miR-133a-3p, miR-122-5p, miR-194-5p, miR-4792, miR-1246, miR-208b-3p, miR-499a-5p and miR-148a-3p		Serum				
miR-34a	Acute or chronic SCI	Serum	.	miRNA-34a is a candidate as a biomarker in acute SCI and can also be used in the monitoring of the neurological remission process.	In human	[94]
Up-regulate: novel_mir39, novel_mir2064, rno-miR-126a-5p, novel_mir603, rno-miR-152-3p, novel_mir51, rno-miR-130a-3p, rno-miR-466b-5p, rno-miR-1188-5p, novel_mir1387	Acute SCI	Serum exosomes	The KEGG pathways targeted by differentially expressed miRNAs included the Wnt signaling pathway, long-term potentiation, axon guidance, ECM-receptor interaction, and focal adhesion.	Serum exosomal miRNAs could be specific and easily detectable diagnostic biomarkers in acute SCI.	In vivo (rat)	[95]
Down-regulate: novel_mir803, novel_mir1945, novel_mir1541, novel_mir513, novel_mir1985, novel_mir4, rno-miR-125b-5p, novel_mir564, novel_mir429						
Up-regulate: novel_mir597, novel_mir1056, rno-miR-3570, rno-miR-500-3p, novel_mir923, novel_mir806, novel_mir829, rno-miR-200b-3p, rno-miR-212-3p, rno-miR-184, miR-485, miR-30b, miR-26b, miR-23b, miR-223, miR-21, miR-211, miR-200c, miR-195, miR-17, miR-133a, miR-125b-1, miR-124, miR-103	Sub-acute SCI	Serum exosomes	The KEGG pathways targeted included metabolic pathways, endocytosis, MAPK signaling pathway, Wnt signaling pathway, cGMP-PKG signaling pathway, Rap1 signaling pathway, purine metabolism, Hippo signaling pathway, focal adhesion, axon guidance, and necroptosis	Serum exosomal miRNAs could be specific and easily detectable diagnostic biomarkers in sub-acute SCI.	In vivo (rat)	[96]
Down-regulate: novel_mir47, rno-miR-99b-3p, novel_mir1034, rno-miR-3588, novel_mir163, rno-miR-369-5p, rno-miR- 3590-5p, rno-miR-301a-3p, rno-miR-1249, miR-672, miR-15b						
miR-19a-3p and miR-19b-3p	Acute or chronic SCI	Plasma	Targets of both miR-19a and miR-19b: PTEN, Rapgef2, voltage-gated potassium channel subunits, Tumor Protein p53 and RUNX3; Targets of miR-19a: SOCS1, MeCP2, RhoB, PPARα, and LRIG1; Targets of miR-19b: FXR1 and STAT3	miR-19a and miR-19b, regulating their common targets, could play important roles in the pathogenesis of neuropathic pain in SCI.	In human	[97]

microRNA: miRNA; spinal cord injury: SCI; Interferon Gamma Receptor 1: IFNGR1; Signal Transduction and Activator of Transcription 2: STAT2; Cytochrome B-245 Beta Chain: CYBB; Nuclear Factor Of Activated T Cells 1: NFATC1; Fc Gamma Receptor IIb: FCGRB; Heme Oxygenase 1: HMOX1; Toll-like receptor 4: TLR4; Hexokinase 2: HK2; long non-coding RNAs: lncRNAs; cerebrospinal fluid: CSF; Kyoto Encyclopedia of Genes and Genomes: KEGG; Mitogen-activated protein kinase: MAPK; cyclic guanosine monophosphate-protein kinase G (cGMP-PKG); Ras-associated protein-1: Rap1; Phosphatase and Tensin Homolog: PTEN; Rap Guanine Nucleotide Exchange Factor2: Rapgef2; Tumor Protein p53: p53; RUNX Family Transcription Factor 3: RUNX3; Suppressor of Cytokine Signaling 1: SOCS1; Methyl-CpG Binding Protein 2: MeCP2; Ras Homolog Family Member B: RhoB; Peroxisome Proliferator Activated Receptor Alpha: PPARα; Leucine Rich Repeats and Immunoglobulin Like Domains 1: LRIG1; FMR1 Autosomal Homolog 1: FXR1; Signal Transduction and Activator of Transcription 3: STAT3.

**Table 2:** Potentials miRNA-based clinical interventions for controlling post-injury symptoms and improving functional recovery.

miRNAs	Period Post-SCI	Treatment	miRNAs Delivery	Targets	Potential Effects	Type of Study	Ref.
miR-7b-3p mimic	Acute SCI 12 h and 3 days	5 nM for 72 h of exposure after 24 h of hydrogen peroxide treatment to induce oxidative stress	Cell transfection	Wipf2	Reduces the percentage of apoptotic nuclei and the expression level of cleaved Casp3, exerting a neuroprotective effect and could promote regeneration modulating plasticity-related genes	in vitro and in vivo	[100]
miR-124 mimics	Acute SCI	20 μM for 14 days after 24 h from SCI	Intrathecal injection	*Tal1*	Promotes neuroprotection and nerve regeneration in SCI rats	in vitro and in vivo	[101]
miR-124	Chronic SCI	-	-	Wnt/β-catenin signaling	miRNA-124 activates Wnt/β-catenin signaling, which in turn appears to be mediated by lncRNA Neat1. It also promoted neural stem cell migration, induced neuron-specific differentiation, resulted in elevated Neat1 expression, accompanied by the functional recovery of locomotion in the mouse model of SCI.	in vitro and in vivo	[102]
Recombinant lentiviral vector containing miR-124	Acute SCI	2 × 10^6^ BMSC transfected with the miR-124-LV vector were transplantation one day after the SCI model	Cell transplantation	*PDXK*	Accelerated the differentiation of BMSCs into neurocytes and promoted the repair of SCI.	In vivo	[103]
miR-448 inhibitor	Acute SCI	40 μM microRNA-448 inhibitor was injected into the injured spinal cord tissue after SCI	Subcutaneously injection	PI3K/AKT/Bcl-2 axis	It protects neurons from apoptosis, induces motor neuron regeneration, and motor recovery, modulating the PI3K/AKT/Bcl-2 pathways.	In vivo	[104]
miR-140 inhibitor	Reactive astrocyte proliferation after SCI	-	Cell transfection	PI3K/AKT/BDNF axis	Increases protein levels of BDNF, p-PI3K, and p-AKT and astrocyte proliferation by the PI3K/AKT pathway	In vitro	[105]
miR-211 inhibitor	Reactive astrocyte proliferation after SCI	-	Cell transfection	PI3K/AKT/BDNF axis	Increases protein levels of BDNF, p-PI3K, and p-AKT and astrocyte proliferation by the PI3K/AKT pathway	In vitro	[106]
miR-21 knockdown	Acute thoracic SCI	contusion followed by a single dose of miR-21 KD vectors (1 × 10^7^ TU)	Subdural injection	TGF-β1, TNF-α and IL-1β BDNF	Reduces the inflammatory response at the damaged spinal cord site reducing TGF-β1, TNF-α, and IL-1β levels, and activity of phosphorylation of AKT. It also promotes motor functional recovery and nerve regeneration increasing BDNF expression.	In vivo	[108]
miR-21- 5p knockdown	SCI	100 nmol/m antagomir-21 for 3 days post-spinal cord contusion	Intrathecal injection.	*SMAD7*	It promotes the pro-fibrogenic activity of TGF-β1 by targeting SMAD7 and improves motor functional recovery.	In vivo	[109]
miR-21a-5p mimics	Acute SCI	48 h after transfection cells were scratched and harvested at 48 h.	Cell transfection	SMAD2/3 phosphorylation	Regulates fibrosis-related gene expression, promotes proliferation, inhibits the apoptosis of spinal fibroblasts, and mediates the SMAD signaling pathway.	in vitro	[110]
miR-21 e miR-199a-3p mimics	SCI	100 nM Biotinylated miR mimics were transfected into DRG neurons	Cell transfection	PTEN and mTOR	Regulates axon growth by modulating PTEN/mTOR pathway.	In vitro	[111]
miR-99b 5p inhibitor		30 µM miR-99b-5p inhibitor	Cell transfection	mTOR	Suppresses SCI-induced neuronal apoptosis and promoted neurite growth reduced after SCI	In vitro	[112]
Lentivirus- miR-29a	Acute thoracic SCI	About 7.5 × 10^5^ TU lentivirus vector -miR-29a	Injection into the lesion site	PTEN	Lentivirus-Mediated Overexpression of miR-29a	In vivo	[113]
miR-29b lentivirus vector	Chronic SCI	0.5 nM miR-29b lentivirus vector were injected 1 h after lesion	Intrathecally injection	-	Ameliorates pathological condition and promotes reductions in neuronal loss and cavity number. Protects neurons from SCI-induced apoptosis, decreasing Bax expression, and improving Bcl-2 expression.	In vivo	[114]
miRNA-29b exosomes	Acute thoracic SCI	One hour after modeling, rats were injected with 200 μg/mL miR-29b exosomes (secreted from miR-29b BM-MSCs) at and with 10^7^ cells/mL of miR-29b BM-MSCs	Intravenously injection through the tail vein	-	Accelerated the motor function of SCI rats, alleviated histopathological damage in spinal cord tissues, and induced neuronal regeneration.	In vivo	[115]
miR-494-modified exosomes	SCI	Co-culture of the constructed miR-494-modified exosome (100–300 pmol) with DRG and rat NR8383 macrophages	Cell transfection	Casp3, Bax, Bcl2, and GFAP	In vitro, inhibits the inflammatory response and neuronal apoptosis. In vivo, promotes regeneration and improves the recovery of behavioral function.	In vitro	[116]
		Rats were treated with 100 μg miR-494-modified exosomes for 24 h for 7 consecutive days after injury.	Intravenously injection through the tail vein			In vivo	
miRNA-26a exosomes	Acute thoracic SCI	PC12 were incubated with miR-26a-overexpressing exosomes (20 μg/mL) for 48 h	Cell transfection	PTEN/AKT/mTOR pathways	Promotes neurofilament regeneration and nerve regeneration in PC12 cells. In vivo ameliorated neuronal and axonal regeneration, and reduced the excessive autophagy, targeting PTEN and mTOR pathways to induce regeneration after SCI.	In vitro	[117]
		Mice received injection of miR-26a exosomes (200 μg) immediately following SCI	Intravenously injection through the tail vein			In vivo	
miR-133b-modified exosomes	Thoracic SCI	-	-	RhoA, GAP-43, GFAP and MBP	Promotes the recovery of neurological function of SCI animals, downregulating RhoA expression levels, and affecting the signaling pathway related to axon regeneration.	In vivo	[118]
Lentivirus encoding miR-133b	Acute SCI	N2A cells were transfected lentivirus-encoding miR-133b	Cell transfection	RhoA	Promotes neurite outgrowth in vitro and improves functional recovery after injury in mice	In vitro	[119]
		Immediately after injury, mice were with 3×10^7^ IU/mL of lentivirus-encoding miR-133b	Injection into the lesion site			In vivo	
miR-133b exosomes	Acute SCI	Rats received 100 μg of miR-133b exosomes 24 h after trauma	Intravenous injection	RhoA, ERK1/2, STAT3 e CREB	Reduces the volume of the lesion and promotes the recovery of neurological function. It protects the neuronal cells from SCI damage and it ameliorates axons regeneration, inhibiting RhoA expression, and increasing phosphorylation of ERK1/2, STAT3, and CREB.	In vivo	[120]
miR-20a	Spinal cord dorsal column lesion	MiR-20a mimics were transfected on the 4th day of cell culture for 24 h	Cell transfection	PDZ-RhoGEF/RhoA/GAP-43	Enhancements of the neurofilament synthesis, neurite regeneration, and sensory conduction function recovery in an animal model of SCI by modulating the PDZ-RhoGE/RhoA/GAP43 axis.	In vitro	[121]
		1.1 µL of recombinant adenovirus of miR-20a was injected in L4-L6 DRG neurons by puncture	Injection into the left L4-L6 DRG neurons			In vivo	
miR-30b	Spinal cord dorsal column lesion	Neurons were transfected with 100 pmol miR-30b agomir for 24 h	Cell transfection	Sema3A/NRP-1/PlexinA1/RhoA/ROCK	Promoted axon growth and ameliorates sensory conductive function by regulating the Sema3A/PlexinA1-NRP-1/RhoA pathway/ROCK.	In vitro	[122]
		1.1 µL miR-30b was injected in L4-L6 DRG neurons by puncture	Injection into the left L4-L6 DRG neurons			In vivo	
miR-220b-3p		6 × 10^8^ TU/mL of lentiviral vector of mi200b-3p were co-transfected into 293 T	Cell transfection	RhoA/ROCK	Induces neuronal stem cells differentiation into neurons to promote motor functional and histological recovery	In vitro	[123]
		Short hairpin mi200b-3p lentiviruses were injected into the injury site	Injection into the lesion			In vivo	
miR-200a	SCI	-	Cell tracking	Brachyury and β-catenin	Determines the identity of neuronal stem cells in the spinal cord during the regenerative process.	In vivo	[124]
miR-22-3p mimics	Spinal cord dorsal column lesion	25 nM	Cell transfection	CBL/p-EGFR/ p-STAT3/GAP-43/p-GAP-43 axis	Increases neurite growth and p-EGFR levels in primary sensory neurons via the p-STAT3/GAP43/p-GAP43 axis	In vitro	[126]
		2.5 nM	Injection into DRG tissue			In vivo	
miR-615 agomir	SCI	Neuronal stem cells were transfected with miR-615 mimics for 48 h	Cell transfection	LINGO-1	Increases neuronal survival and proliferation, enhances axonal extension and myelination, and improves recovery of hindlimbs motor functions	In vitro	[127]
		miR-615 agomir (20 nmol/mL) was delivered(1 μL/h) into the lesion site of SCI rats for 3 days through osmotic mini-pumps implanted at T12/T13 immediately after SCI	Intrathecal administration			In vivo	
miR-125b	Cervical SCI	-	-	*JAK1* and *STAT1*	Promotes the repair and regeneration	In vivo	[128]
miR-372 inhibitor	SCI	-	-	*KIF3B* and *NOSIP*	In human neuronal stem cells rescued the IL-1β-induced impairment as shown by significant improvements in behavioral assessments in SCI rats.	In vivo and in vitro	[130]
adeno-associated virus-expressing miR-210	Acute SCI	Microinjection of 20μL adeno-associated virus-expressing miR-210 into the rat spinal cord at the T10-11 interspace	Subarachnoid injection	PTP1B	Protects neurons and improves neurologic function scores. Decreases activities of Casp3 and 8, increases vessel count in the spinal cord and regulates serum levels of inflammation-related cytokines.	In vivo	[131]

WAS/WASL interacting protein family member 2: Wipf2; Caspase 3: Casp3; pyridoxal kinase: PDXK; small mother against decapentaplegic: SMAD; knockdown: KD; tumor necrosis factor α: TNFα; transforming growth factor β: TGF-β; interleukin-1β: IL-1β; protein kinase B: AKT; mechanistic target of rapamycin: mTOR; Ras homolog gene family member A: RhoA; Rho-associated protein kinase: ROCK; bone marrow mesenchymal stem cells: BM-MSCs; growth-associated protein 43: GAP-43; glial fibrillary acidic protein: GFAP; myelin basic protein: MBP; Extracellular regulated kinase 1/2: ERK1/2; cAMP response element-binding protein: CREB; dorsal root ganglia: DRG; RhoA and PSD-95/Dlg/ZO-1-Rho guanine nucleotide exchange factor: PDZ-RhoGEF; Semaphorin 3A: Sema3A; Neuropilin 1: NRP-1; Brain derived neurotrophic factor: BDNF; B-cell lymphoma: Bcl-2; Bcl-2-associated X protein: Bax; Phosphoinositide 3-kinase: PI3K; epidermal growth factor: EGF; phosphorylated-epidermal growth factor receptor: p-EGFR; Casitas B-lineage lymphoma: CBL; phosphor- Growth-Associated Protein 43: p-GAP43; LRR and Ig domain-containing NOGO receptor-interacting protein 1: LINGO-1;: Janus Kinase 1: JAK1; Kinesin family member 3B: KIF3B; nitric oxide synthase interacting protein: NOSIP; Protein tyrosine phosphatase 1B: PTP1B.

## Data Availability

Not applicable.
